# *α*-Glycerol monolaurate promotes tight junction proteins expression through PKC/MAPK/ATF-2 signaling pathway

**DOI:** 10.3389/fnut.2025.1598991

**Published:** 2025-07-31

**Authors:** Siyu Dai, Cunjie Li, Dagang Li, Hong Wang

**Affiliations:** 1 Guangdong Province Engineering Research Center for Antibody Drug and Immunoassay, College of Life Science and Technology, Jinan University, Guangzhou, Guangdong, China; 2 Institute of Animal Science, Guangdong Academy of Agricultural Sciences, Guangzhou, Guangdong, China

**Keywords:** intestinal epithelial cells, proteomic analysis, *α*-glycerol monolaurate, tight junction proteins, PKC/MAPK/ATF-2 signaling pathway

## Abstract

**Introduction:**

This study investigates the effects of *α*-GML on intestinal epithelial tight junction (TJ) protein expression and its molecular mechanisms. Recognizing the critical role of TJ proteins in intestinal barrier function and the potential of *α*-GML to enhance this barrier, we employ the IPEC-J2 cell model. Our aim is to validate the regulatory impact ofα-GML on TJ protein expression and elucidate the underlying signaling pathways, thereby offering new strategies for intestinal health maintenance.

**Methods:**

Utilized Data-Independent Acquisition (DIA) analysis to identify optimal targets of *α*-GML in modulating Tight Junction (TJ) protein expression. Treated cells with specific inhibitors of PKC and MAPK to assess their role in TJ regulation by *α*-GML. Co-treated cells with the MAPK inhibitor SCH772984 and α-GML to study the effects on p-ATF-2 expression. Evaluated the effects of SCH772984 and ATF-2 overexpression on the protein expression levels of phosphorylated ATF-2, ZO-1, and OCLN.

**Results:**

Revealed that *α*-GML’s modulation of TJ proteins might involve the PKC/MAPK signaling pathway, leading to ATF-2 phosphorylation. Both PKC and MAPK inhibitors reduced TJ protein expression (*p* < 0.05, *p* < 0.01 or *p* < 0.001), indicating their involvement in *α*-GML’s regulation. SCH772984 counteracted α-GML-induced upregulation of p-ATF-2 (*p* < 0.05), suggesting MAPK’s role in this process. Identified potential ATF-2 binding sites on ZO-1 and OCLN promoters. ATF-2 significantly enhanced ZO-1 promoter activity (*p* < 0.001). SCH772984 reduced phosphorylated ATF-2, ZO-1, and OCLN levels (*p* < 0.05 or *p* < 0.01), while ATF-2 overexpression rescued this decrease (*p* < 0.05 or *p* < 0.01), confirming ATF-2’s role in TJ protein upregulation via the MAPK pathway.

**Discussion:**

Our study indicated that *α*-GML enhanced the expression of TJ proteins through the PKC/MAPK/ATF-2 pathway, thereby enhancing the barrier function of intestinal epithelial cells.

## 1 Introduction

Intestinal epithelial cells are seamlessly integrated through a intricate network of intercellular junctions, encompassing gap junctions, desmosomes, tight junctions (TJs), and adherence junctions (AJs) (1). TJ proteins, which include Claudins, OCLNs, junctional adhesion molecules (JAMs), and zonula occluden-1 (ZO-1), serve as vital intercellular adhesion complexes within epithelial and endothelial barriers, forming the essential paracellular barrier (2). Intestinal TJ damage allows harmful substances to pass through, triggering immune responses and inflammation, potentially leading to diseases. Upregulating the expression of TJ, however, is beneficial for restoring and maintaining intestinal health (3, 4). Dong et al. demonstrated that mannose alleviated dextran sodium sulfate (DSS)-induced colitis by protecting the intestinal barrier and strengthening tight junctions (5). Li et al. has demonstrated that chitosan oligosaccharide can significantly ameliorate lipopolysaccharide (LPS)-induced intestinal epithelial barrier damage by upregulating the levels of TJ proteins in an extracellular signal-regulated kinase 1/2 (ERK1/2)-dependent manner (6).

Protein kinase C (PKC) is a family of serine- and threonine-specific protein kinases and mediates many cellular processes in a tissue-specific manner, significantly influencing a wide range of agonists, including growth factors, neurotransmitters, and hormonal stimuli, thereby governing key signaling processes (7). The PKC/mitogen-activated protein kinases (MAPK) pathways are recognized as classic cellular signaling pathways that are intimately linked to the regulation of TJs in epithelial cells. However, the effect of PKC/MAPK on TJ expression is reduced or increased, depending on the cell type and condition (8). Jo et al. demonstrated that oxyresveratrol activates the PKC and MAPK pathways, markedly enhancing the levels of TJ-related genes and proteins, reducing monolayer permeability, and increasing transepithelial electrical resistance, thereby reinforcing the integrity of the intestinal barrier (9). Similarly, Li et al. found that tea polyphenols inhibit the toll-like reporter 4 (TLR-4)/MAPK/PKC-myosin light chain kinase (MLCK) signaling pathway, thereby reducing the release of inflammatory cytokines and blocking the decrease in *β*-catenin and OCLN expression induced by *Actinobacillus pleuropneumoniae* in newborn pig tracheal epithelial cells (10).

*α*-Glycerol monolaurate (α-GML), a monoglyceride consisting of glycerol and lauric acid, possesses diverse pharmacological and biological activities and is used both as a food additive and in medicinal applications (11, 12). The US Food and Drug Administration (FDA) has designated GML as a generally recognized as safe food emulsifier (13). Numerous studies have indicated that GML significantly improves the function of the intestinal epithelial barrier. Kong et al. demonstrated that the restoration of intestinal injury in LPS-challenged broilers is predominantly attributed to the potent effects of GML supplementation, which significantly enhanced the expression of TJ proteins and activated the adenosine monophosphate-activated protein kinase (AMPK)/nuclear factor-erythroid 2-related factor 2 (Nrf2) pathway (14). He et al. GML treatment suppressed the activation of the MAPK and nuclear factor kappaB (NF-κB) signaling pathways, enhanced the expression of intestinal TJ proteins ZO-1, OCLN, and Claudin-1, and mitigated tissue damage, thereby relieving the macroscopic symptoms in mice suffering from DSS-induced colitis (15). Despite these findings, the impact of *α*-GML on the expression of intestinal epithelial TJs remains largely unexplored. Therefore, this study aimed to assess the role and molecular mechanism of α-GML in the expression of TJ proteins using IPEC-J2 cells as an *in vitro* model.

## 2 Materials and methods

### 2.1 Reagents and materials

Porcine small intestinal epithelial cell line (IPEC-J2 cells, third-generation primary cells) were purchased from Huatuo Biotechnology Co.

### 2.2 Cell culture and treatment

IPEC-J2 cells were grown in dulbecco’s modified eEagle’s medium (DMEM, Gibco, China) supplemented with 10% fetal bovine serum (FBS, Gibco, China) under an atmospheric condition of 95% O_2_ /5% CO_2_ at 37°C in a humidified incubator. The culture medium was refreshed every 2 days during cell growth.

### 2.3 FITC-dextran assay

The cell concentration was adjusted to 1.5 × 10^5^ cells/mL, then 1 mL of cell suspension was transferred to the upper chamber of the transwell. When the cell density reached 80%, replaced the upper and lower chambers with medium containing 0.01 mM *α*-GML (Kevin, China) and incubated the cells for 24, 36, 48 and 60 h, respectively. The upper chambers were then replaced with medium containing 1 mg/mL fluorescein isothiocyanate-dextran (FITC-dextran, Aladdin, China), and the lower chambers were replaced with phosphate buffered saline (PBS). After incubation in a CO_2_ incubator protected from light for 1 h, the PBS in the lower chamber was collected, and the relative fluorescence content of the PBS was measured by using an enzyme marker at an excitation wavelength of 493 nm and an emission wavelength of 518 nm.

### 2.4 Cell viability assay

The cell concentration was adjusted to 4 × 10^4^ cells/mL, add 100 μL of cell suspension to each well of a 96-well plate, and incubate the cells in DMEM complete medium (10% FBS) until the density reaches about 50%, and then replace the DMEM complete medium with DMEM containing 0, 0.05, 0.03, 0.01, 0.008, 0.005, 0.003, and 0.001 mM *α*-GML and 1% dimethyl Sulfoxide (DMSO, MP Biomedicals, China). Complete medium was used to culture the cells. After culturing the cells in α-GML working solution for 24, 36, 48 and 60 h, 10 μL of cell counting kit-8 (CCK8, NCM Biotech, China) reagent was added to each well, and the cells were incubated in a cell culture incubator protected from light for 1 h. Then the absorbance value was detected at 490 nm using an enzyme marker, and the absorbance value of each well minus the absorbance value of the blank-well medium could represent the cell activity.

### 2.5 Total RNA extraction and quantitative real-time PCR (qRT-PCR)

At approximately 80% confluence, the cells were exposed to a complete medium supplemented with 0.01 mM *α*-GML for varying durations, or subjected to various inhibitors across different groups for a continuous period of 48 h. Total RNA was extracted using total RNA isolation kit (NCM Biotech, China), and the cDNA was synthesized using the PrimeScript first-strand cDNA synthesis kit (Novoprotein, China). The qRT-PCR was performed using the SYBR qPCR SuperMix Plus kit (Novoprotein, China) at 95°C for 1 min, followed by 40 cycles of 95°C for 20 s and 60°C for 1 min. Reaction system was listed in Table 1. The levels of gene expression were quantified using the 2^−ΔΔCt^ method, and the GAPDH gene was used as the reference gene. The primer sequences were listed in Table 2.

**Table 1:** Reaction system for qRT-PCR.

Reagents	Dosage
2 × SYBR qPCR SuperMix Plus	10 μl
Upstream primers	0.2 μM
Downstream primers	0.2 μM
Template	2 μL
RNase free water	To 20 μL

**Table 2:** Gene primers sequence used for qRT-PCR.

Primers	Forward (5′-3′)	Reverse (5′-3′)	Source of sequence
ZO-1	TGGGAACAGCACACAGTGAC	GCTGGCCCTCCTTTTAACAC	10.1016/j.toxlet.2023.07.007
OCLN	ATGCTTTCTCAGCCAGCGTA	AAGGTTCCATAGCCTCGGTC	10.1016/j.ecoenv.2022.114006
Claudin-1	AGATTTACTCCTACGCTGGTGAC	GCAAAGTGGTGTTCAGATTCAG	10.1016/j.ecoenv.2022.114006
Claudin-2	TTGTGACAGCAGTTGGCTTC	TCATGCCCACCACAGAGATA	10.1007/s00011-021-01468-9
ATF-2	TACAAGTGGTCGTCGG	CGGTTACAGGGCAATC	10.3892/etm.2021.10015
GAPDH	ATTTGGCTACAGCAACAGGG	CAACTGTGAGGAGGGGAGA	10.1016/j.tiv.2019.104700

### 2.6 Western blot assay

When the cell density reached about 80%, cells were incubated in complete medium containing 0.01 mM *α*-GML for different times, or treated with different inhibitors (groups: control, *α*-GML, 0.5 nM Staurosporine, α-GML + 0.05 nM Staurosporine, α-GML + 0.5 nM Staurosporine, α-GML + 1 nM Staurosporine, α-GML + 2 nM Staurosporine, 0.5 μM SCH772984, α-GML + 0.1 μM SCH772984, α-GML + 0.5 μM SCH772984, α-GML + 1 μM SCH772984, α-GML + 2 μM SCH772984) for consecutive 48 h. Total protein from cells was extracted using RIPA lysis buffer (NCM Biotech, China). The appropriate amount of protein was separated by 10% sodium dodecyl sulfate-polyacrylamide gel electrophoresis, transferred to PVDF membranes (Millipore, USA) which then were blocked with 5% non-fat dried milk at 37°C for 1 h. Then the membranes were incubated with primary antibodies (1:1000, Bioss, China) overnight at 4°C and then incubated with secondary antibodies conjugated with goat anti-rabbit IgG (H&L) HRP (1:2000, Affinity, China) for 1 h at room temperature. ImageJ software was employed for the quantification of protein bands. For each group, the grayscale values of GAPDH were utilized as an internal control to normalize the data. Each experiment was conducted in triplicate to ensure the reliability and reproducibility of the results.

### 2.7 Analysis of functional and pathway enrichment

When cell density reached approximately 80%, cells were incubated in complete medium containing 0.01 mM *α*-GML (groups: control, DMSO, α-GML). Cells were then lysed with RIPA lysis buffer and stored at −80°C. The cell samples were then sent to Guangzhou Genedenovo Biotechnology Co., Ltd. for further processing and preparation, and high-throughput, high-precision data acquisition and analysis were conducted on the Omicsmart online platform^1^ using Data-Independent Acquisition (DIA) proteomics technology. The proteins with *p* value<0.05 and absolute value of fold change (FC) greater than 1.2-fold were considered as differentially expressed proteins (DEPs). Finally, functional annotation and pathway analysis of the identified differentially expressed proteins were performed to reveal their potential biological significance and mechanisms of action. Gene Ontology (GO), KEGG and Reactome pathway analysis was used to understand the biological processes and pathways that enriched IPEC-J2 cells after *α*-GML treatment.

### 2.8 Dual-luciferase reporter assays

The cell concentration was adjusted to 1.5 × 10^5^ cells/mL and were seeded in a 12-well plate for 12 h. The plasmid of dual luciferase reporter assay system and ATF-2 overexpression was co-transfected into the cells using Lipofectamine 2000 (Invitrogen, USA). After 48 h, the transfected cells with luciferase reporter genes were lysed centrifuged for 3 min. The luciferase activity (firefly catalyzes luciferin at a wavelength of 560 nm and renilla luciferase at a wavelength of 465 nm in the supernatant) was measured with the substrates of dual luciferase reporter assay kit (Yeasen Biotechnology, China) using CLARIOstar microplate readers (BMG LABTECH, Germany).

### 2.9 Statistical analysis

Statistical analysis was performed using IBM SPSS 26.0 and Graph pad Prism 9.5 analysis software. The LSD-*t* test was used for two-by-two comparisons between groups, and one-way ANOVA was used for comparisons between multiple groups. Significant differences were found when *p* < 0.05.

## 3 Results

### 3.1 *α*-GML promotes the expression of TJ genes and proteins in IPEC-J2 cells

Cells treated with various concentrations of α-GML for different times showed no significant effect on proliferation at 24 h (*p* > 0.05), but a significant increase was observed at 48 h with 0.01 mM α-GML, which was also effective at enhancing proliferation at 60 h (*p* < 0.05, *p* < 0.001) (16). This concentration was chosen for further experiments, where it significantly reduced FITC-dextran flux through IPEC-J2 cells at 36, 48, and 60 h, indicating improved barrier function (*p* < 0.05 or *p* < 0.001) (16). qRT-PCR and western blot assays revealed that 0.01 mM *α*-GML significantly elevated the mRNA and protein levels of ZO-1 and OCLN at 36, 48, and 60 h (*p* < 0.05, *p* < 0.01), with no impact on the expression levels of Claudin-1 and Claudin-2 (*p* > 0.05) (16). Collectively, these results preliminarily suggested that 0.01 mM α-GML significantly up-regulated the expression of ZO-1 and OCLN, and reduced the paracellular permeability of IPEC-J2 cells. This effect is most pronounced after 48 h, thus 48 h has been established as the standard treatment duration for subsequent studies.

### 3.2 Proteomic analysis and identification of ATF-2 as a potential target in regulatory pathways

**Figure 1:**
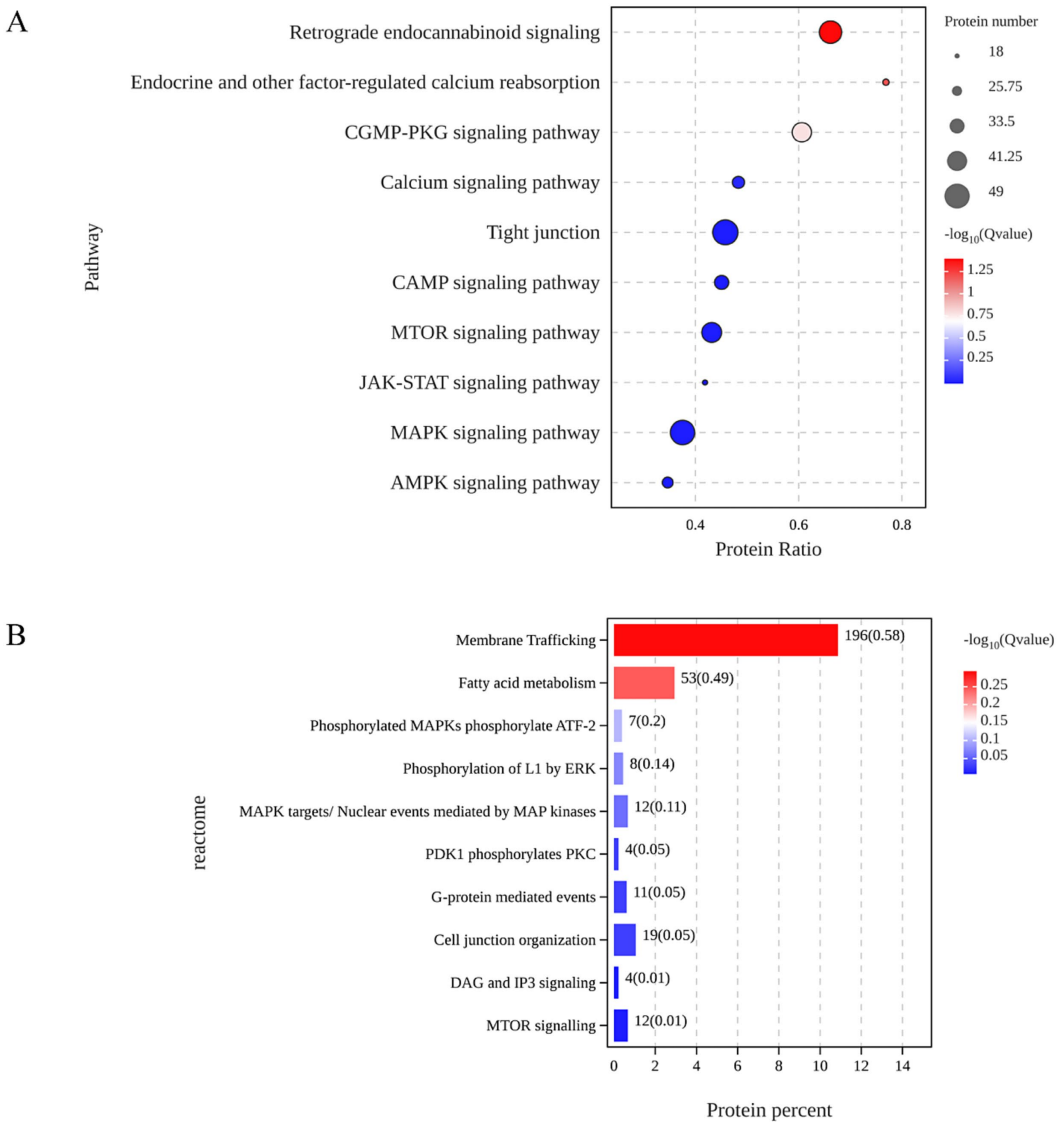
ATF-2 was indicated to be the potential target of α-GML in up-regulation of TJ expression based proteomic analysis. **(A)** KEGG analysis on the common targets. **(B)** Signal pathway enrichment by Reactome analysis.

Data-independent acquisition (DIA)-based quantitative proteomics was employed to identify and quantify proteins for untargeted proteomics experiments (17). This analysis identified a total of 3,692 differentially expressed proteins (DEPs), with 1,598 proteins upregulated and 2094 proteins downregulated in the *α*-GML-treated group compare with the DMSO group (Supplementary Figure 1A). The result of GO analysis indicated that α-GML affected biological processes, molecular functions and cellular components in cells. And the top three biological processes were cellular processes, metabolic processes, and biological regulation (Supplementary Figure 1B). Further insights from the KEGG pathway analysis implicated the MAPK signaling pathway as a potential mechanism related to TJs, based on the prevalence of common molecular targets and the significance of the *p-*values, suggesting that *α*-GML regulates the expression of TJ proteins through the PKC/MAPK pathway(Figure 1A). The result of Reactome analysis showed that MAPK might phosphorylate activating transcription factor 2 (ATF-2) (Figure 1B). It is reported that ATF-2 had close relevance to intestinal epithelial barrier (18, 19). Therefore, ATF-2 may be a potential transcription factor for up-regulating TJ expression.

### 3.3 *α*-GML promotes the expression of TJ genes and proteins through PKC/MAPK signaling pathway

**Figure 2:**
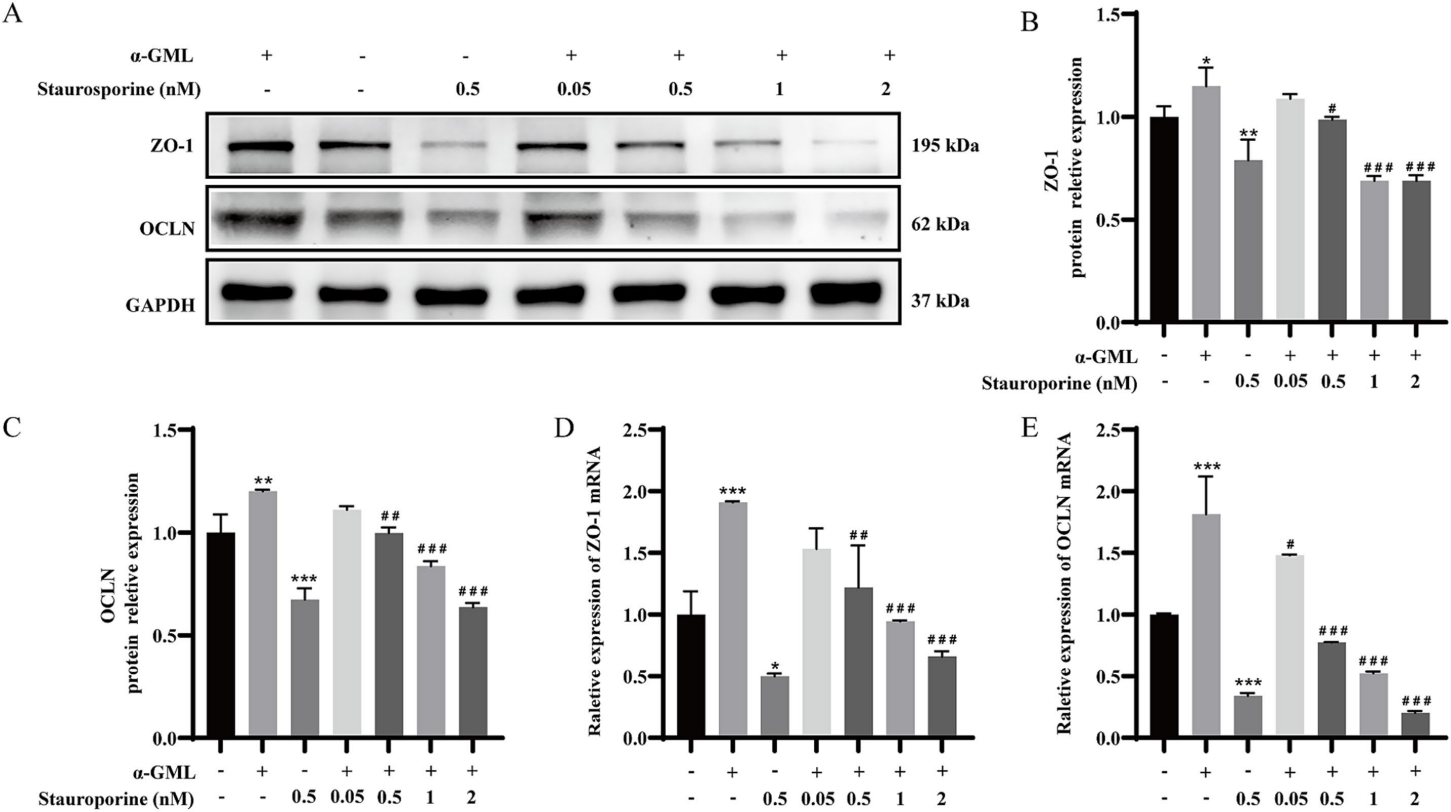
α-GML promoted TJ expression through activation of PKC. **(A)** The effects on ZO-1 and OCLN protein expression of different concentrations of Staurosporine with or without α-GML. **(B,C)** Western blot quantification of ZO-1 and OCLN. **(D,E)** The mRNA expression of TJ proteins among different groups by qRT-PCR. Values are shown as means ± SEs, *n* = 3. *p < 0.05, **p < 0.01, ***p < 0.001 vs. the DMSO group. ^#^*p* < 0.05, ^##^*p* < 0.01, ^###^*p* < 0.001 vs. the α-GML treated group.

**Figure 3:**
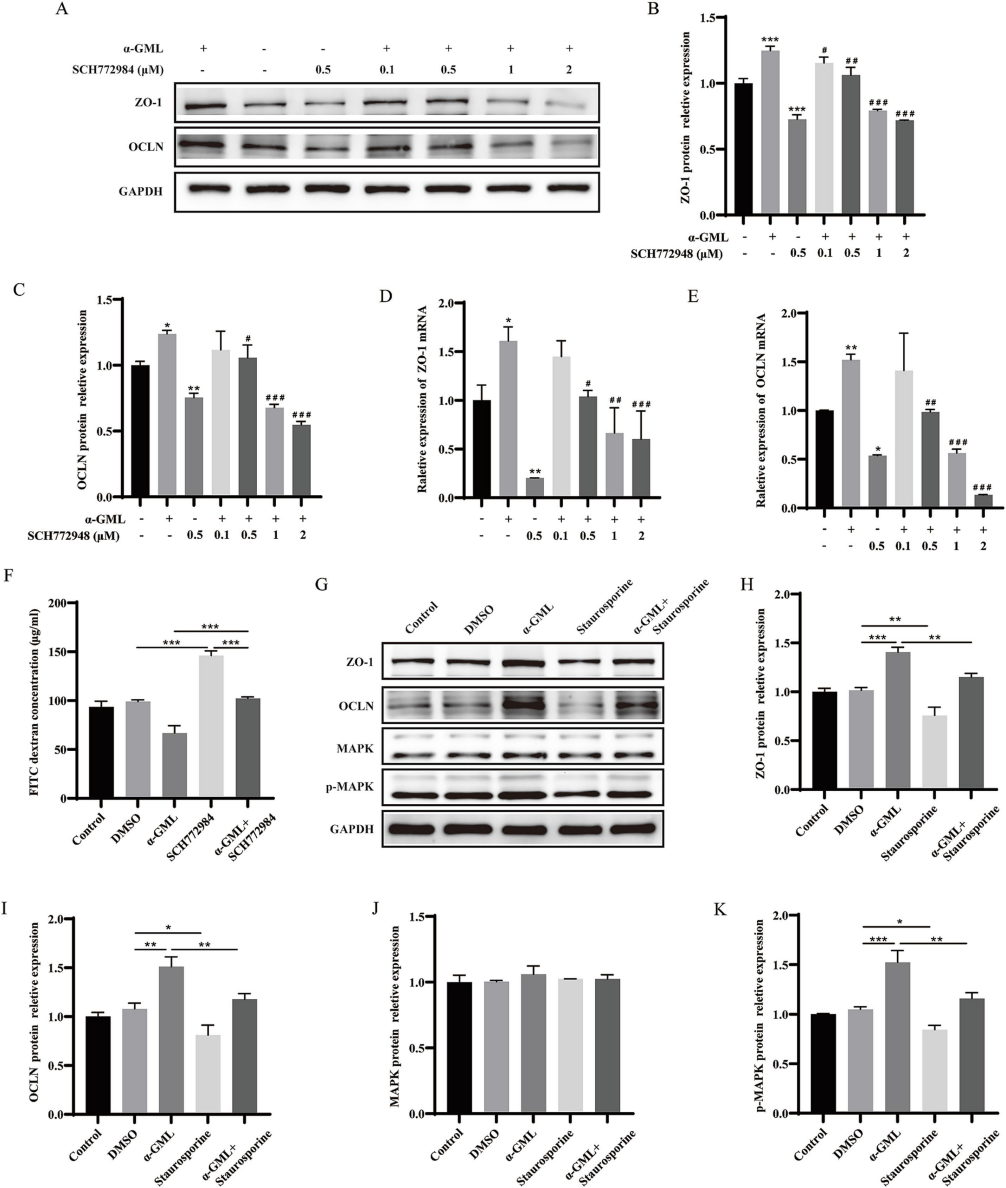
α-GML promoted expression of TJ through PKC/MAPK signaling pathway. **(A–C)** The effects of different concentrations of SCH772984, with or without α-GML, on the expression of TJ proteins by western blot and quantification. **(D,E)** The mRNA expression of TJ proteins among different groups by qRT-PCR. **(F)** The results of FITC-dextran flux of cells treated with SCH772984 and α-GML. **(G–K)** Effects of Staurosporine and α-GML on the protein expression of phosphorylated and non-phosphorylated MAPK as well as TJ by western blot and quantification. Values are shown as means ± SEs, *n* = 3. *ns*, *p* > 0.05, *p < 0.05, **p < 0.01, ***p < 0.001 vs. the DMSO group. ^#^*p* < 0.05, ^##^*p* < 0.01, ^###^*p* < 0.001 vs. the α-GML treated group.

To investigate the role of PKC in TJ proteins expression, we treated cells with the PKC inhibitor Staurosporine during culture and assessed changes at both the transcriptional and protein levels. Western blot analysis demonstrated a marked reduction in ZO-1 and OCLN protein expression in cells treated with 0.5 μM Staurosporine, contrasting with a significant increase in the *α*-GML-treated group compared to the DMSO control (*p* < 0.01 or *p* < 0.001). In the Staurosporine and α-GML co-treated groups, protein expression was progressively inhibited with increasing inhibitor concentration (Figures 2A–C, *p* < 0.05, *p* < 0.01 or *p* < 0.001). And the trend of TJ mRNA expression in the experimental group was consistent with the trend of TJ proteins expression (Figures 2D,E, *p* < 0.05, *p* < 0.01 or *p* < 0.001). We further treated the cells with MAPK inhibitor SCH772984, and the results were consistent with the trend observed using the PKC inhibitor (Figures 3A–E, *p* < 0.05, *p* < 0.01 or *p* < 0.001). In addition, the results showed that SCH772984 exposure significantly increased paracellular permeability (*p* < 0.001), indicating that SCH772984 impaired the cell barrier function. Co-treatment of SCH772984 and *α*-GML showed that α-GML functionally reversed the increase of the concentration of FITC-dextran induced by SCH772984 (Figure 3F, *P* < 0.001).

Since MAPK is downstream of PKC, cells are treated with PKC inhibitor Staurosporine to detect the phosphorylation level of MAPK protein by western blot assay. The results indicated that Staurosporine significantly decreased the expression of p-MAPK (*p* < 0.05 or *p* < 0.01). However, co-treatment with *α*-GML reversed the reduction in p-MAPK expression induced by Staurosporine (*p* < 0.01), with no significant differences observed in total MAPK expression across all groups (Figures 3G–K, *p* > 0.05). These findings suggest that *α*-GML’s upregulation of TJ expression is indeed associated with the PKC/MAPK pathway.

### 3.4 α-GML facilitates the expression of TJ proteins by activating the transcription factor ATF-2

**Figure 4:**
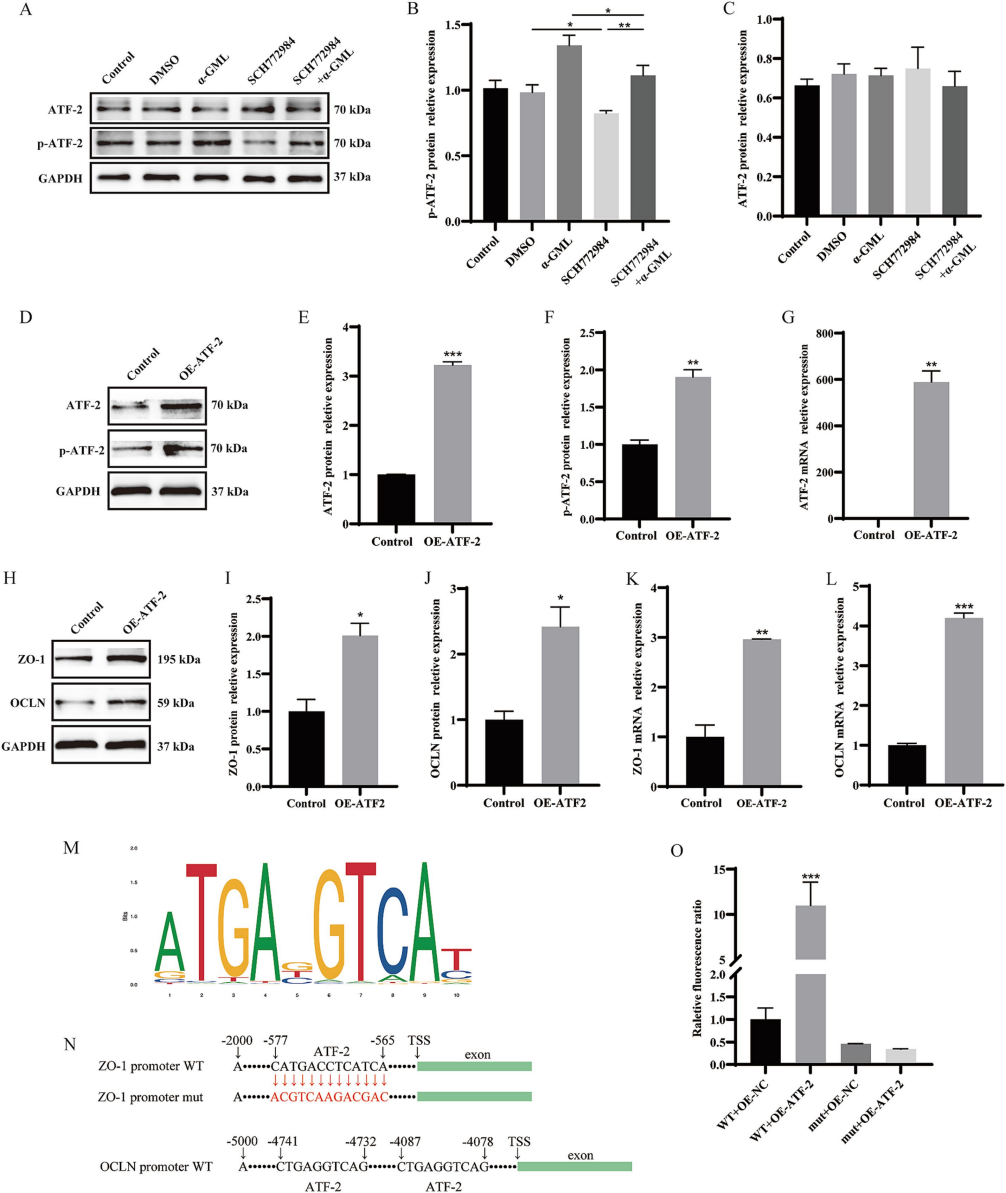
Modulation of ATF-2 phosphorylation and TJ proteins levels by a MAPK inhibitor and ATF-2 overexpression. **(A–C)** Effects of SCH772984 and α-GML on the protein expression of phosphorylated and non-phosphorylated ATF-2 by western blot and quantification. **(D–G)** Effect of OE-ATF-2 on expression of p-ATF-2 and ATF-2 by western blot and qRT-PCR. **(H–L)** Effect of OE-ATF-2 on expression of TJ proteins by western blot and qRT-PCR. **(M)** The motif of ATF-2 transcription factor binding to DNA. **(N)** The ATF-2 binding site on the ZO-1 and OCLN promoter sequence. **(O)** The mutant promoter activity of ZO-1 analyzed by Dual-Luciferase reporter assays. Values are shown as means ± SEs, *n* = 3. ns, *p* > 0.05, *p < 0.05, **p < 0.01, ***p < 0.001 vs. the Control group.

According to the above results, we proposed the mechanisms by which *α*-GML might promote the expression of TJ proteins by affecting MAPK/ATF-2 signaling pathway. The results showed that the treatment of α-GML significantly up-regulated the phosphorylation level of ATF compared with DMSO group (*p* < 0.05). Co-treatment of MAPK inhibitor SCH772984 and α-GML showed that SCH772984 functionally reversed the increase of the expression of p-ATF-2 induced by α-GML (*p* < 0.01). Expression of ATF-2 protein was not obviously different in all groups (Figures 4A–C, *p* > 0.05). To investigate the role of ATF-2 in modulating the expression of TJ proteins, we transfected cells with the plasmid designed for ATF-2 overexpression. The results showed that the expression level of ATF-2 and p-ATF-2 was significantly up-regulated (Figures 4D–G, *p* < 0.01, *p* < 0.001). Similarly, the expression level of TJ proteins was up-regulated after overexpressed with ATF-2 in cells (Figures 4H–L, *p* < 0.05, *p* < 0.01, *p* < 0.001).

Given that ATF-2 is a transcription factor, we postulated that ATF-2 may activate TJ transcription by directly binding to the promoter, thereby enhancing protein expression. We predicted the presence of ATF-2 binding sites upstream of the ZO-1 and OCLN transcription start sites (TSS) utilizing the JASPAR database available at https://jaspar.elixir.no/ (Figure 4M). Our findings identified a consensus ATF-2 binding site within the ZO-1 TSS, located between −565 bp and −577 bp (CATGACCTCATCA) with a relative profile score threshold 98.54%. What’s more, the OCLN promoter sequence harbors two distinct ATF-2 binding sites, located at −4,078 bp to −4,087 bp and −4,732 bp to −4,741 bp (CTGAGGTCAG), respectively, with a relative profile score threshold of 89.72%. To further validate the role of the ATF-2 binding sequence in ZO-1 promoter activity, a site-directed mutagenesis was performed to mutate the ATF-2 binding site (CATGACCTCATCA to CGTCAAGACGAC) within the ZO-1 promoter region. The resulting plasmid, along with the ATF-2 overexpression plasmid, was co-transfected into the cells using a dual luciferase reporter assay system. We found that the ATF-2 overexpression plasmid caused a significant increase in ZO-1 promoter activity (*p* < 0.001). The mutation of the ATF-2 binding site inhibited the increase in ZO-1 promoter activity mediated by ATF-2 overexpression (Figures 4N,O, *p* > 0.05).

**Figure 5:**
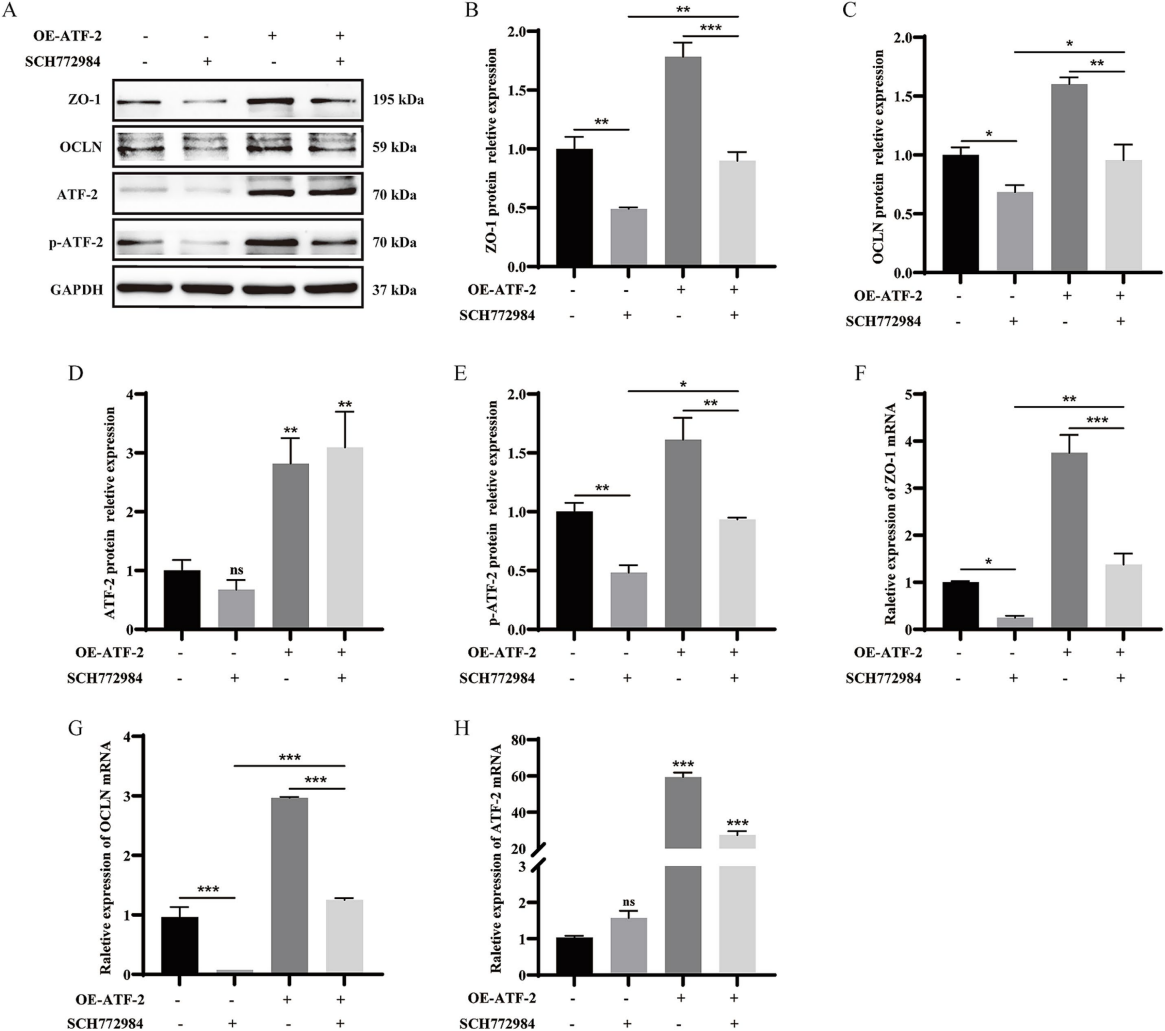
α-GML facilitated the expression of ZO-1 and OCLN through MAPK/ATF-2 signaling pathway. **(A–E)** Co-treated with SCH772984 and ATF-2 overexpression plasmid to detect TJ and p-ATF-2 expression by western blot assay and quantification. **(F–H)** The mRNA expression of TJ proteins among different groups by qRT-PCR. Values are shown as means ± SEs, *n* = 3. *p < 0.05, **p < 0.01, ***p < 0.001 vs. the DMSO group.

To delineate the mechanism of TJ expression mediated by ATF-2, we investigated the interplay between MAPK and ATF-2. Cells were then pretreated with SCH772984 and ATF-2 overexpression plasmid, western blot assay and qRT-PCR was used to detect TJ and ATF-2 expression. The findings indicated that SCH772984 markedly reduced the protein expression of p-ATF-2, ZO-1, and OCLN (*p* < 0.05 or *p* < 0.01), whereas the expression level of ATF-2 remained unchanged in comparison to the control group (*p* > 0.05). Concurrent treatment with SCH772984 and OE-ATF-2 demonstrated that OE-ATF-2 was able to functionally reverse the decrease in p-ATF-2 and TJ proteins expression induced by SCH772984 in IPEC-J2 cells (Figures 5A–E, *p* < 0.05, *p* < 0.01), yet the expression level of ATF-2 still did not exhibit a significant change (*p* > 0.05). The mRNA expression trends for ATF-2 and TJ were consistent with the protein expression patterns (Figures 5F–H, *p* < 0.05, *p* < 0.01 or *p* < 0.001). The results confirmed that *α*-GML treatment elevated the phosphorylation status of the transcription factor ATF-2 by activating MAPK, subsequently increasing the expression of TJ proteins.

## 4 Discussion

GML represents a safe and efficacious substitute for antibiotic growth promoters, offering broad-spectrum antibacterial, antiviral, and immunomodulatory effects that can enhance livestock and poultry performance and health (20). Previous studies have shown that incorporating GML into the diet can strengthen the functional and structural integrity of the intestinal barrier by optimizing the expression and arrangement of TJ proteins. Liu et al. the dietary of GML in broilers has been observed to augment their performance metrics, optimize intestinal morphology, and elevate muscle amino acid profiles, predominantly through the orchestration of the gut microbiota’s community dynamics, functional capacity, and metabolic profiles (21). Cui et al. showed that GML supplementation in a low-protein, antibiotic-free diet for weaning piglets increased the expression of TJ proteins in the jejunum, thereby enhancing intestinal barrier health, promoting growth, and potentially preventing infections from bacterial endotoxins and toxic macromolecules (22). However, the exact mechanism by which GML enhances the intestinal barrier remains to be elucidated. Our prior studies have indicated that dietary *α*-GML increases the protein expression of OCLN and ZO-1 in the jejunum, promoting the development of the intestinal barrier, and enhancing the immunity of partridge chicks (23). In the current study, we investigated the mechanism by which α-GML promotes the expression of TJ proteins, revealing that α-GML treatment activates the transcription factor ATF-2 via the PKC/MAPK signaling pathway, thereby upregulating the expression of TJ proteins.

MAPKs, including ERK1/2, c-Jun N-terminal kinases (JNK), p38 MAPK, and other ERK-related kinases, regulate a wide array of cellular processes such as transcriptional regulation, proliferation, differentiation, and apoptosis, mediating various biological outcomes (24, 25). Yong et al. demonstrated that tea tree oil upregulated the expression of TJ proteins and reduced intestinal mucosal barrier permeability, improving barrier integrity through the MAPK1/2 signaling pathway (26). He et al. showed that Huanglian Ganjiang ameliorates colitis symptoms in mice, which may be associated with the restoration of TJs and intestinal barrier integrity and function, potentially through the suppression of the APOC1-JNK/P38 MAPK signaling pathway (27). These studies suggest that MAPK-related signaling pathways are involved in the regulation of TJ proteins expression. Additionally, MAPK pathways modulate protein expression by phosphorylating ATF-2, a basic/leucine zipper (bZIP) motif-containing member of the leucine zipper family of DNA-binding proteins, with research primarily centered on its roles in cancer and inflammation (28, 29). There are also studies suggesting that ATF-2 might be involved in the regulation of TJs. Al-Sadi et al. demonstrated that the IL-1β-mediated enhancement of intestinal tight junction permeability, both *in vitro* and *in vivo*, was governed by the activation of p38 kinase, which in turn activated ATF-2, and by ATF-2’s regulatory influence on MLCK gene activity (19). Miao et al. discovered that the disturbance in the expression and distribution of TJ proteins induced by deoxynivalenol might be mediated by the activation of the p38/ATF-2/MLCK signaling pathways (30). However, no studies to date have established a direct link between ATF-2 and the expression of intestinal epithelial TJ proteins.

DIA analysis revealed that the optimal targets of *α*-GML in modulating TJ proteins expression might involve the PKC/MAPK signaling pathway, leading to the phosphorylation of ATF-2. We treated cells with the inhibitors of PKC and MAPK separately and found that the two inhibitors reduced the expression levels of TJs, indicating that PKC/MAPK is involved in the regulation of TJs by α-GML. Subsequent co-treatment with the MAPK inhibitor SCH772984 and α-GML revealed that SCH772984 effectively counteracted the α-GML-induced upregulation of p-ATF-2 expression levels. Since ATF-2 is a transcription factor, we postulated that it activates TJ transcription by directly binding to the promoters, thereby promoting protein expression. We identified potential ATF-2 binding motifs on the ZO-1 and OCLN promoters using the JASPAR database. Furthermore, through the dual-luciferase reporter system, we confirmed that ATF-2 significantly enhanced the promoter activity of ZO-1. Pretreatment with SCH772984 and ATF-2 overexpression plasmid demonstrated that SCH772984 reduced the protein expression levels of phosphorylated ATF-2, ZO-1, and OCLN, while OE-ATF-2 could rescue the decrease in expression induced by SCH772984, indicating that *α*-GML treatment activated the transcription factor ATF-2 through the MAPK signaling pathway, leading to the upregulation of TJ proteins expression.

In summary, our study demonstrates that α-GML enhances intestinal epithelial barrier function by upregulating TJ proteins through the PKC/MAPK/ATF-2 pathway. While previous studies have established *α*-GML’s ability to improve intestinal barrier function (14, 15), the underlying molecular mechanisms remained unclear. Our work provides three key advances: (1) identification of the specific PKC/MAPK/ATF-2 signaling cascade activated by α-GML, (2) demonstration that ATF-2 phosphorylation directly activates ZO-1 and OCLN transcription through promoter binding - a novel finding extending beyond ATF-2’s known roles in inflammation (19), and (3) mechanistic distinction from previous reports of general MAPK/PKC involvement in TJ modulation (9, 10). These findings not only elucidate α-GML’s mode of action but also support its potential application in livestock feed for intestinal health. While our functional studies clearly establish this signaling pathway, future structural work could further characterize direct binding interactions, as suggested by recent reports of monoglyceride-PKC (31) and laurate-MAPK (32) interactions.

## Data Availability

The datasets presented in this study can be found in online repositories. The names of the repository/repositories and accession number(s) can be found in the article/Supplementary material.

## Footnotes

^1^ https://www.omicsmart.com/
