# Correction: α-Glycerol monolaurate promotes tight junction proteins expression through PKC/MAPK/ATF-2 signaling pathway

**DOI:** 10.3389/fnut.2025.1732647

**Published:** 2025-11-17

**Authors:** Siyu Dai, Cunjie Li, Dagang Li, Hong Wang

**Affiliations:** 1 Guangdong Province Engineering Research Center for Antibody Drug and Immunoassay, College of Life Science and Technology, Jinan University, Guangzhou, Guangdong, China; 2 Institute of Animal Science, Guangdong Academy of Agricultural Sciences, Guangzhou, Guangdong, China

**Keywords:** intestinal epithelial cells, proteomic analysis, α-glycerol monolaurate, tight junction proteins, PKC/MAPK/ATF-2 signaling pathway

There was a mistake in Image 2 and Image 3 in the **Supplementary material** as published. The original supplementary material inadvertently included these two figures which were not cited in the manuscript and should not have been published. The corrected supplementary material file should contain one Table and one Image.

The original article has been updated.

