# Supplementary figures and images for: α-Glycerol monolaurate promotes tight junction proteins expression through PKC/MAPK/ATF-2 signaling pathway
Source: Front Nutr. 2025 Jul 31;12:1598991. doi: 10.3389/fnut.2025.1598991 (PMC12350310; doi:10.3389/fnut.2025.1598991)

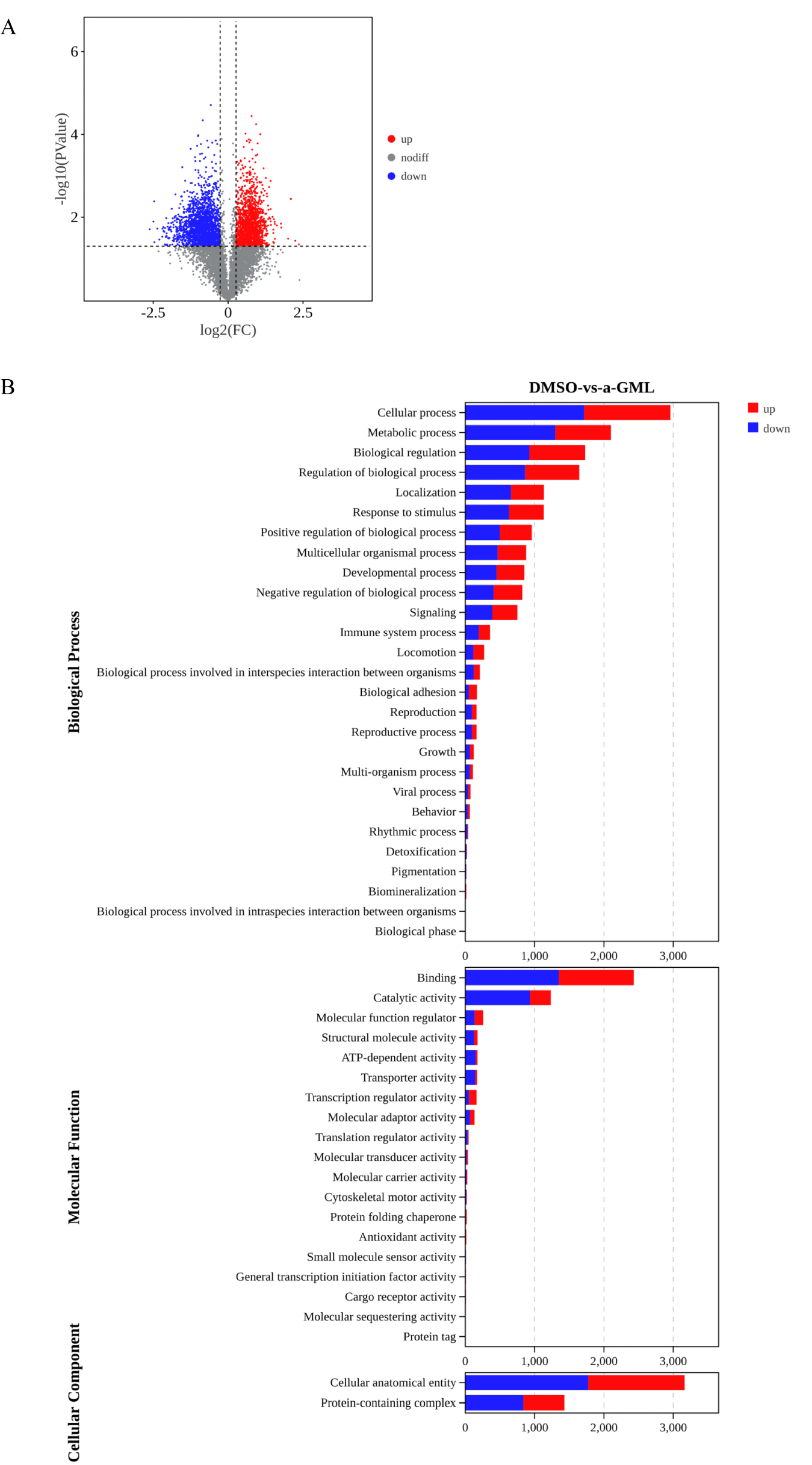

Supplement: Supplementary file 1 [file Image_1.tif]
