# Supplementary material for: α-Glycerol monolaurate promotes tight junction proteins expression through PKC/MAPK/ATF-2 signaling pathway
Source: Front Nutr. 2025 Jul 31;12:1598991. doi: 10.3389/fnut.2025.1598991 (PMC12350310; doi:10.3389/fnut.2025.1598991)
Supplement: Supplementary file 2 [file Table_1.docx]

**Supplementary Material of Table 1.**

Image 1. Proteomic analysis in the treatment of α-GML. (A)Volcano plot showing the differentially expressed proteins between DMSO group and α-GML group. (B) GO analysis on the common targets between DMSO group and α-GML group.
